# Mechanistic insights into MET exon 14 skipping mutations and their role in tumor progression

**DOI:** 10.1042/BST20253091

**Published:** 2025-09-09

**Authors:** Promita Ghosh, Isabella Pecora, Morag Park

**Affiliations:** 1 Department of Biochemistry, McGill University, Montréal, QC, Canada; 2 Goodman Cancer Institute, McGill University, Montréal, QC, Canada; 3 Department of Medicine, Division of Experimental Medicine, McGill University, Montreal, Quebec, Canada; 4 Department of Oncology, McGill University, Montréal, QC, Canada

**Keywords:** drug resistance, MET, NSCLC, targeted therapy, mutation

## Abstract

The MET receptor tyrosine kinase is a pivotal regulator of cellular survival, motility, and proliferation. Mutations leading to skipping of exon 14 (METΔex14) within the juxtamembrane domain of MET impair receptor degradation and prolong oncogenic signaling, contributing significantly to tumor progression across multiple cancer types. METΔex14 mutations are associated with aggressive clinical behavior, therapeutic resistance, and poor outcomes. Next-generation sequencing from both tissue and liquid biopsies has significantly improved the detection frequency of METΔex14 in lung and other cancers. However, clinical trials targeting METΔex14 have rendered partial responses and mixed outcomes due to the lack of a comprehensive mechanistic understanding of METΔex14 regulation and a diverse mutational landscape. This review synthesizes current knowledge on the mechanistic basis of METΔex14-driven oncogenesis, including alterations in receptor dynamics, downstream signaling perturbations, genomic alterations underlying this mutation, and mechanisms of acquired therapeutic resistance. We further discuss the clinical implications of these insights and highlight future research directions essential for optimizing targeted therapies.

## Introduction

Aberrant activation of receptor tyrosine kinase (RTK)-mediated signaling pathways is a hallmark of many cancers, promoting tumor initiation, progression, and metastasis [1,2]. Among these RTKs, the *MET* proto-oncogene, which encodes the hepatocyte growth factor (HGF) receptor, has emerged as a prominent oncogenic driver across diverse human malignancies, including lung, brain, and gastrointestinal tumors among others [3,4].

The MET RTK was initially discovered as a transforming oncogene in human osteogenic sarcoma cell lines exposed to the chemical carcinogen *N*-**met**hyl-*N*'-nitro*-N*-nitrosoguanidine (MNNG), from which it derives its name ‘*MET*’ [5,6]. Physiologically, MET promotes a ‘cell-scattering’ epithelial-to-mesenchymal transition phenotype, a dynamic process that breaks down epithelial cell–cell junctions and promotes cell motility during early development, tissue repair, and regeneration in adults [7,8]. Genetic alterations in MET are associated with dysregulated cell transduction programs and oncogenic transformations and, in general, are associated with poor outcomes [3,9,10].

Mutations leading to skipping of exon 14 (MET∆ex14) was first reported in 2003 in small-cell lung cancer followed by its discovery in non-small cell lung cancer (NSCLC) in 2005 [11,12]. MET exon 14 skipping mutations are the most commonly reported oncogenic *MET* mutations, and >200 distinct genomic alterations have been identified by comprehensive cancer genome profiling [13,14]. Molecular abnormalities in and around exon 14, including point mutations, insertions, and deletions (indels), can disrupt essential consensus sequences such as splice acceptor and donor sites, polypyrimidine tract, and branch sites and give rise to this splicing variant, which results in the loss of 141 base pairs, leading to a 47-amino acid in-frame deletion within the juxtamembrane domain (JXD) [9,15].

MET∆ex14 mutations are more commonly described in NSCLC cases and usually occur in the absence of other known driver mutations such as *EGFR, KRAS,* or *ALK* [9,14,15]. MET amplification (METamp) through copy gain of the *MET* gene on chromosome 7q31 [16] has been reported to occur mostly in advanced-stage NSCLC patients, where the *MET∆ex14* allele was found to be selectively amplified over the wildtype (WT) *MET* allele [9,17,18]. MET∆ex14 mutations also occur in ~14% of secondary glioblastoma multiforme (sGBM), lower frequencies in primary GBM (pGBM), low-grade GBM (LGG), and gastroesophageal cancer [19,20].

MET∆ex14 mutations in NSCLC primarily affect the elderly population (median age ~>70 years) compared with other oncogenic drivers and are linked to poor prognosis [21–23]. The FDA has approved capmatinib [24] and tepotinib [22], highly selective and potent oral MET inhibitors for MET∆ex14-amplified tumors that have demonstrated significant clinical benefits in both treatment-naïve and previously treated patients with advanced, metastatic NSCLC. Since MET∆ex14 is considered an actionable biomarker in NSCLC, other tyrosine kinase inhibitors (TKIs), antibodies, and combination therapies are being investigated for efficacy and safety. However, a major challenge to clinical benefit in patients with MET∆ex14 is diverse mechanisms of acquired resistance to MET TKI monotherapy [25,26].

## Biology and activation mechanisms of METΔex14

MET is a 190-kDa transmembrane RTK composed of an entirely extracellular 50-kDa α-chain and a 140-kDa β-chain joined through disulfide bonds [27] predominantly expressed in epithelial cells but can also be expressed by endothelial cell populations and macrophages [28]. Its physiological ligand, HGF, also known as scatter factor, is primarily produced by mesenchymal cells [29,30] as well as macrophages [31]. HGF has two distinct binding sites for MET and two HGF molecules act to bridge two MET molecules, which activates the kinase, enabling trans-phosphorylation of twin tyrosines (Y1234/1235) within the catalytic loop in the kinase domain [32]. The induction of MET catalytic activity is critical for triggering subsequent phosphorylation of Y1349 and Y1356 in the carboxy (C)-terminus of MET forming an SH2 domain recognition motif (Y1349VHVX3Y1356VNV) for downstream signaling [33,34]. The SH2 domain recruits downstream adaptor and scaffold proteins to activate a multilayered MET signaling network [3,10,35].

A key regulatory tyrosine, Y1003, located within exon 14, when phosphorylated, engages with the CBL SH2 domain, leading to polyubiquitination of MET and its subsequent degradation by the lysosome [36–38]. A MET mutant carrying a substitution of Y1003 with a phenylalanine (Y1003F) is not ubiquitinated and shows an enhanced transforming phenotype when expressed in both fibroblast and epithelial cells exposed to HGF [39]. The loss of exon 14 preserves the reading frame and, upon HGF stimulation, results in delayed degradation and oncogenic activation of METΔex14 [38,39]. In a recent study, cells stably expressing METΔex14 or MET Y1003F were found to promote similar transcriptional programs upon HGF stimulation for 24 h [40]. This supports that loss of the CBL-binding site is a key event that renders METΔex14 oncogenic.

MET exon 14 also harbors a caspase cleavage site. Under conditions of apoptotic stress, MET can be cleaved at the ESVD1002 site in the JXD and the DNADDEVD1380 site near the C-terminus. This cleavage event leads directly to the inactivation of the MET receptor [41].

Another regulatory site located in exon 14 of MET is S985, which, when phosphorylated by PKC, confers an inhibitory action on MET activation by down-regulating the kinase activity of MET downstream [33,42]. Engineering a chimeric construct (*TPR-juxtaMET*) by inserting MET exon 14 into the oncogenic fusion protein TPR-MET, which lacks the JXD and key regulatory residues S985 and Y1003, significantly reduced cell proliferation, anchorage-independent growth, and fibroblast invasion compared with full-length MET [43].

In summary, each of these negative regulatory sites is functionally distinct in regulating MET stability. Mutations causing the substitution of Y1003, which abolish the CBL-binding site, are a rare alteration when compared with the incidence of METΔex14 [44]. This suggests that, despite sharing transcriptional signatures with MET Y1003, METΔex14 can confer additional advantage to the tumor beyond decreased MET ubiquitination.

## Oncogenic signaling regulated by METΔex14

Upon HGF-mediated activation of MET, various signaling cascades, including the PI3K/AKT, RAS/MAPK, and STAT3 pathways, are activated. This occurs through the recruitment of adaptor proteins, GRB2 and SHC, which bind to MET Y1356 [45–47]. MET predominantly signals through a scaffold protein GAB1, which is recruited to MET Y1356 through its indirect interaction with GRB2 SH3 domains [48–50] and enhanced through engagement of 13 amino acids within the GAB1 MET binding domain that interact with Y1349 present in the multisubstrate binding site on the MET C-terminus [51]. Once recruited, GAB1 becomes tyrosine phosphorylated by MET providing binding sites for downstream signaling proteins, including the p85 subunit of PI3K, SHP2, PLCγ, and Crk through their SH2 domains [48,51]. GRB2 recruits SOS, a guanine nucleotide exchange factor for RAS, subsequently leading to transient activation of the MAPK signaling pathway [52,53], whereas GAB1 recruitment of SHP2 leads to a more prolonged MAPK pathway activation [54]. MET activation also leads to the recruitment and activation of the transcription factor STAT3, which, upon phosphorylation, translocates to the nucleus and drives the expression of important cell survival signals [55].

**Figure 1:**
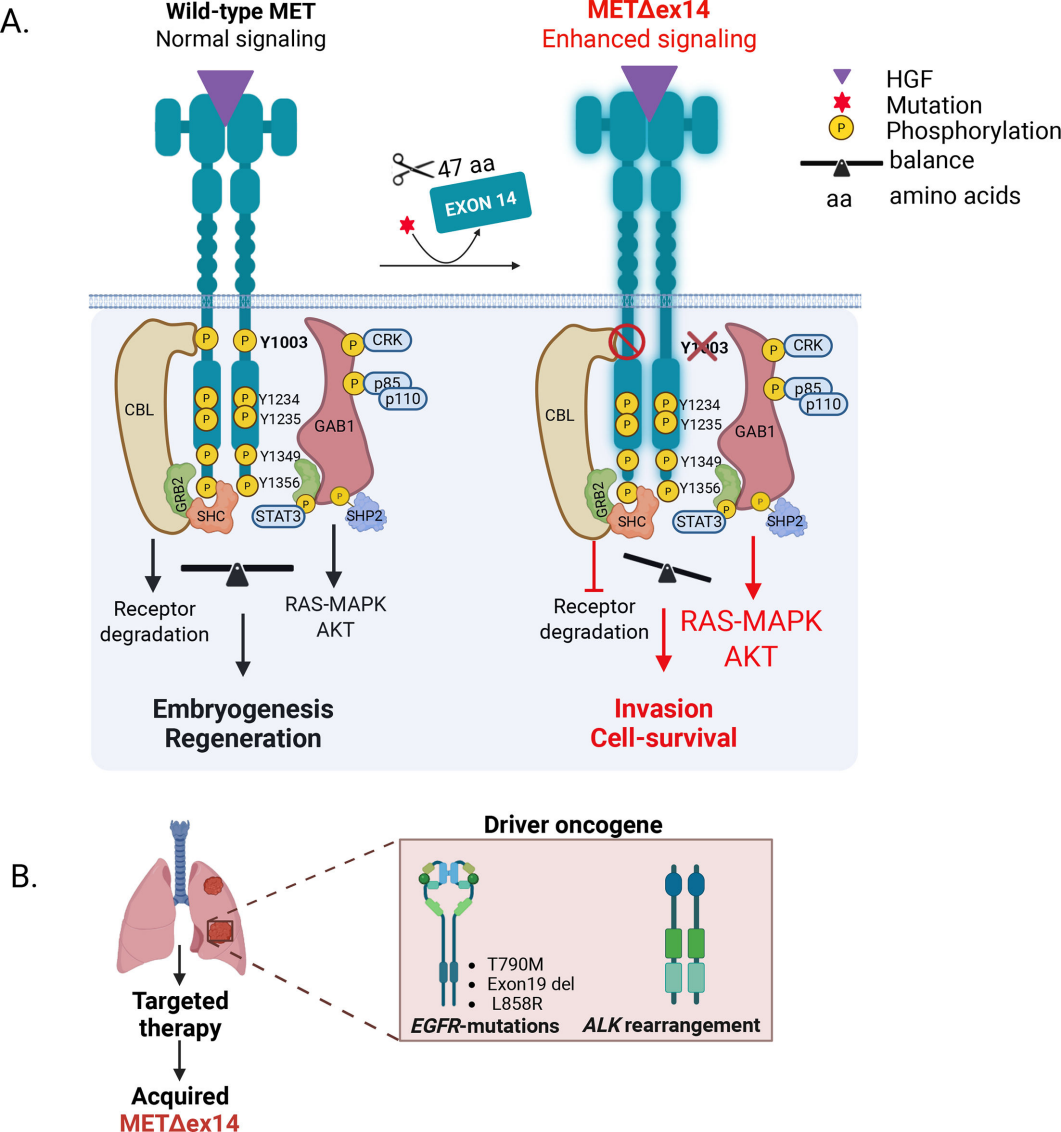
METΔex14 as an oncogenic driver and a mechanism of resistance. **A.** Upon HGF stimulation wildtype (WT) MET receptors dimerize, leading to phosphorylation of tyrosines Y1234 and Y1235 in the tyrosine kinase domain, which promotes further receptor phosphorylation and recruitment of adaptor proteins for downstream signaling. Exon 14 in MET contains Y1003, a regulatory site that recruits CBL for MET degradation (left). In METΔex14, loss of exon 14 removes the CBL-binding site, preventing MET degradation. HGF stimulation of METΔex14 leads to elevated MAPK and AKT signaling, enhancing transcription of genes involved in motility and adhesion (right). **B.** METΔex14 as a mechanism of resistance in EGFR-mutant and ALK rearranged NSCLC. HGF, hepatocyte growth factor; METΔex14, mutations leading to skipping of exon 14;

METΔex14 exhibits altered trafficking, with reduced sorting to early and late lysosomes [56]. Due to impaired HGF-induced receptor degradation caused by the absence of Y1003, METΔex14 exhibits prolonged activation of downstream signaling pathways, including PI3K/AKT and RAS/MAPK [Figure 1A]. Compared with MET WT, METΔex14 cells injected into the mouse tail vein displayed increased tumor growth and metastasis to the lungs and brain, potentially driven by enhanced extracellular matrix remodeling, cytoskeletal reorganization, and focal adhesion formation [56]. The METΔex14 mutation, in the absence of other driver mutations, has also been shown to be capable of transforming normal lung epithelial cells (16HBE) and inducing tumor formation [57]. Dose-response experiments demonstrated that METΔex14+ cells exhibit increased sensitivity to lower levels of HGF, establishing altered kinetics compared with WT MET [58].

A key signaling difference between MET WT and METΔex14 is the enhanced activation of the RAS–MAPK pathway [59,60]. Upon HGF stimulation, METΔex14 cell lines exhibit increased phosphorylation of RAS-dependent effectors (MEK1/2 and ERK1/2) but not RAS-independent effectors (PI3K and STAT3) when compared with MET WT. At the transcriptional level, RNA-seq analysis of METΔex14 patient-derived cell lines treated with HGF identified up-regulation of multiple genes within the HALLMARK_KRAS_UP gene set, including *SERPINE1, MMP1*, and *NRP1*, which are linked to metastasis and invasion, as well as *ELK3, ETV1*, and *TMEM158*, which are associated with cell growth and survival. Notably, KRAS knockdown selectively reduced cell viability of METΔex14 cells, highlighting a unique dependency on this pathway [59].

## Immune modulation

The advent of immune checkpoint inhibitors (ICIs), designed to disrupt PD-1/PD-L1 or CTLA-4/CD80 (CD86) interactions, has provided an effective strategy to potentiate T cell-mediated anti-tumor immunity. Although a significant subset of METΔex14 lung cancers exhibits high PD-L1 expression, the clinical efficacy and response to immunotherapy (IO) remain contentious [61,62]. In a cohort of 110 German patients, treatment with chemotherapy (CHT) and IO, either as monotherapies or in combination, revealed that the CHT–IO combination conferred superior clinical efficacy irrespective of PD-L1 expression, compared with either modality alone [63]. Genomic profiles of 122 (3.2%) MET∆ex14 and 70 (1.8%) METamp out of 3821 NSCLC tumors were compared on the Strata Select assay, and MET∆ex14 displayed elevated expression of immune regulatory genes associated with immune tolerance, such as *CTLA4, PD-1, LAG3*, *IDO1, TIGIT*, and *HAVCR2*, compared with WT or METamp tumors [64]. Further comparisons between METΔex14, METamp, and MET WT tumors revealed that METΔex14 tumors exhibit the lowest tumor mutational burden (TMB) and neoantigen tumor burden, which may contribute to their variable response to ICIs [61]. Identifying clonal neoantigens generated from MET exon 14 skipping mutations could be effective in patients with acquired resistance to TKIs and treated with ICIs, as has been shown previously in other driver mutations, such as *EGFR* [65].

Although comprehensive characterization of immune cell infiltration in METΔex14 tumors remains limited, emerging data indicate a significantly enriched immune microenvironment relative to MET WT tumors, with pronounced up-regulation of IFN-γ and T cell inflammation gene signatures. METΔex14 tumors have been reported to harbor diverse immune infiltrates, including M1 and M2 macrophages, CD4^+^ and CD8^+^ T lymphocytes, Tregs, and dendritic cells [44,66], indicative of an immunological complex yet heterogeneous tumor immune microenvironment that may contribute to variable responses to IO and potentially poorer prognosis.

### Clinicopathological features and genomic landscape of METΔex14 tumors

### NSCLC

Several studies have assessed the heterogeneity of METΔex14 mutations by comparing the clinical landscape, mutation profiles, and pathological features in patients across different stages and treatment status. Though there is some variability in the distribution of histology subtypes across different studies due to unique patient demographics, the most frequently reported histology associated with METΔex14+ tumors is adenocarcinoma (~>60%) followed by squamous cell (~≤10%), adenosquamous (~2.5%), sarcomatid (~2.5–4%) and large cell (<1%) [44,67,68]. Historically, most METΔex14 alterations in the functional splice sites of exon 14 have been found in the splice donor and acceptor sites, with base substitutions being the most frequent type of mutation [44,68,69]. In a multicenter retrospective analysis study of 148 patients with METΔex14 NSCLC, 71 of whom had advanced to stage IV disease, the most common sites of metastases were lymph nodes (67%) and lung (53%), followed by pleural/pericardial metastases or malignant effusions (51%), bone (49%), and brain (37%) [70]. Offin et al. reported that CNS metastases, including leptomeningeal disease, occurred in more than one-third of patients with METΔex14 lung cancers, increasing the risk of death at least by two-fold compared with patients with no brain metastasis [71].

Co-alterations in *TP53* are the most frequently reported concurrent alteration in METΔex14 patients [44,68,72]. Marks et al. reported that *TP53* mutations showed enrichment in METΔex14+ non-squamous (nSq) histology compared with METΔex14− nSq tumors, suggesting distinct associations of *TP53* mutations with histology [73]. Co-amplifications of *MDM2, HMGA2*, and *CDK4* have been reported by independent groups in METΔex14 patients; however, *MDM2* amplification and *TP53* alterations are mutually exclusive in most cases [44,68]. Sequencing 711 METΔex14+ patients demonstrated that copy number amplifications in *MDM2, HMGA2,* and *CDK4* are more prevalent in METΔex14+ tumors independent of histology, while amplifications in *LGR5, WIF1, LRIG3*, and *MET* were increased only in METΔex14+ nSq tumors compared with METΔex14− tumors [73].

Querying three datasets of METΔex14 NSCLCs, which include Guardant360 (July 2019 to July 2020), GenePlus (both circulating tumor DNA [ctDNA] and tissue February 2017 to April 2020), and the VISION trial ctDNA cohort (NCT02864992), it was reported that the frequency of METamp co-occurring with METΔex14 was 8.4% in Guardant360, 13.8% in VISION, and 7.6% in GenePlus, respectively [69]. However, copy-number amplifications of *MET* were not significantly coincident with *MDM2/CDK4* amplification [17]. The co-occurrence patterns for genes and alterations in both METΔex14 ctDNA and tissue samples are similar, but detection of both *MDM2* amplification and *MET* co-amplification is less common in liquid than tissue samples of METΔex14 NSCLC [68]. Co-amplification of *MET* with METΔex14 is associated with stage IV NSCLC and resulted in a 24-fold increase in *MET* expression compared with a three-fold increase in expression with METΔex14 alone [44],[18]). Patients with METΔex14 NSCLC with or without concurrent METamp show significantly lower copy number alterations and varied TMB distributions [67,74].

As has been established by many groups in independent cohorts, METΔex14 tumors rarely harbored other driver molecular alterations, thereby making METΔex14 the *de novo* driver oncogene in these tumors [13,60]. Interestingly, studies that focused on cohorts with advanced-stage or stage IIIB**/**IV METΔex14 patients reported the presence of activating mutations in *KRAS* with G12C mutations being the most frequently occurring type [68,72,74]. Cell-free DNA profiling from 289 advanced-stage METΔex14 patients revealed 10 co-occurring alterations, of which *NF1, KRAS,* and *NRAS*, key components of the RAS–MAPK pathway, displayed a higher frequency of co-occurrence in METΔex14 patients when compared with an independent cohort of *EGFR-*mutated NSCLC [72]. Comparing METamp and METΔex14 cohorts directly, Castiglione et al. reported similar but not identical genomic variabilities in the two groups – copy number gains in *MYC, CCND1,* and *TERT* and amplification in *MDM2, CDK4,* and *DYRK2* were detected majorly in METΔex14 patients, whereas *MYC* high-level amplifications, *MYC* copy number gains, and a recurrent low copy number gain in *CCND1* and *CCND2* were found in METamp patients [74]. However, it remained unclear whether METamp patients showed a higher incidence of carrying an amplified METΔex14 allele over the WT MET allele, as has been observed in other studies [9,17]. Jamme et al. reported advanced-stage NSCLC patients with METΔex14 carrying alterations in the PI3K pathway, including *PTEN* loss and *PIK3CA* mutations, which can be associated with resistance to MET-TKI [75]. Comparable results were noted by Liu et al. in a retrospective analysis of 31 patients with METΔex14, where *PIK3CA* mutations were present in 9.7% of the patients, and out of which half of them showed disease progression with MET TKI therapy, indicating primary resistance to MET TKIs [76].

### GBM

Independent studies have reported elevated levels of HGF and MET in human gliomas compared with control brain tissue, with expression levels correlating to tumor grade [77,78]. Integrated genomic and/or transcriptomic data from 188 sGBM patients demonstrated significant enrichment of METΔex14 in sGBM compared with LGG or pGBM and weredetected both independently and in oncogenic fusion with *PTPRZ1 (PTPRZ1–MET*). Seven out of 11 patients with METΔex14 showed a concurrent METamp, and all 11 METΔex14 patients displayed *CDKN2A* mutations. Most of these METΔex14 patients also showed concurrent mutations in *IDH1 (Isocitrate Dehydrogenase 1)* and *ATRX (Alpha-Thalassemia/Mental Retardation, X-linked),* which are both strong prognostic factors in glioma [79], along with mutations in *TP53* [20].

### MetΔex14 cell lines

Currently, there are two patient-derived cell lines available, which display METΔex14, namely NCI-H596, isolated from a male with lung adenocarcinoma (LUAD), and a complex hypertriploid gastric carcinoma cell line, Hs746T [80,81]. NCI-H596 harbors non-amplified METΔex14 and an E545K mutation in exon 9 of *PIK3CA* [82] and shows resistance to type I MET inhibitors [75,83,84] likely due to the bypass hyperactivation of the PI3K pathway. Combined inhibition of type I MET inhibitors and PI3K has shown promise in promoting cell growth arrest in H596 and other MET exon 14 skipping-engineered cells [75,85]. However, a unique type II MET inhibitor, glesatinib, was able to inhibit HGF-induced MET phosphorylation, as well as growth in H596, thereby exhibiting an alternate mechanism to overcome resistance to classical type I MET inhibitors [86].

Hs746T displays a homozygous mutation in the splice acceptor site of MET exon 14, as well as concurrent METamp. In contrast with H596, Hs746T shows a strong response to both class I and II MET inhibitors, as well as to combinations of MET inhibitors with other drugs [17,25,85,86]. More detailed studies with larger cohorts and clinical trials will be necessary to clearly define if patients with METamp NSCLCs with or without METΔex14 have a survival advantage and show increased sensitivity to MET TKIs over patients with just METΔex14.

## MetΔex14 screening – the present and future

METΔex14 represents a clinically significant group of mutations, which are complex and exhibit heterogeneous sequence composition, making their detection technically challenging [87]. A wide range of of testing methods is being used to capture the diverse spectrum of MET exon 14 mutations, including IHC, FISH, PCR-based assays, and Sanger sequencing, but next-generation sequencing (NGS) is the most prevalent method to detect METΔex14 in routine testing [88]. RNA-based NGS testing in independent evaluations displayed stronger sensitivity in identifying METΔex14 cases that otherwise remained undetectable by DNA-based testing [89,90]. In an evaluable cohort of 5570 NSCLC patients, concurrent RNA-NGS and DNA-NGS detected 18.6% more patients harboring MET exon 14 skipping alterations compared with DNA-NGS alone [91].

Although NGS is rapid and a more stringent method to detect METΔex14, high cost, complex data interpretation, and longer turnaround times can affect critical clinical decisions [92]. Molecular testing that shortens the turnaround time is of cytopathological importance. An example is the Idylla GeneFusion Assay developed by Biocartis (Mechelen, Belgium), which is an automated cartridge-based, qualitative PCR instrument that detected the presence of METΔex14 with 94% accuracy along with some other actionable markers in only 3 hours [93,94]. Similar results were observed in a multicenter study using the Idylla GeneFusion technology, which successfully established high sensitivity/specificity of 92.5%/99.6% for METΔex14 mutations [95].

Traditionally, tumor tissue has been the preferred biospecimen for molecular testing. Since the publication of the first liquid biopsy statement by the International Association for the Study of Lung Cancer in 2018 [96], the ‘plasma first’ approach has gained clinical relevance as a standard of care and is the preferred choice in cases of inadequate tissue, a common bottleneck in lung cancer [97]. In a phase II, multicenter study, patients with NSCLC harboring *MET* or *MET* exon 14 skipping alterations receiving tepotinib demonstrated higher response rates detected by the plasma NGS test at baseline, making this minimally invasive procedure a desirable alternative to tumor biopsies [98]. Jürgen et al. demonstrated high concordance between the tumor tissue-based RT-PCR assay and the plasma-based F1LCDx for METΔex14 detection, with similar capmatinib clinical activity in METΔex14 + NSCLC patients identified by either method, supporting broader adoption of liquid biopsy testing [99].

## Therapy resistance mechanisms and combinatorial treatment

**Figure 2:**
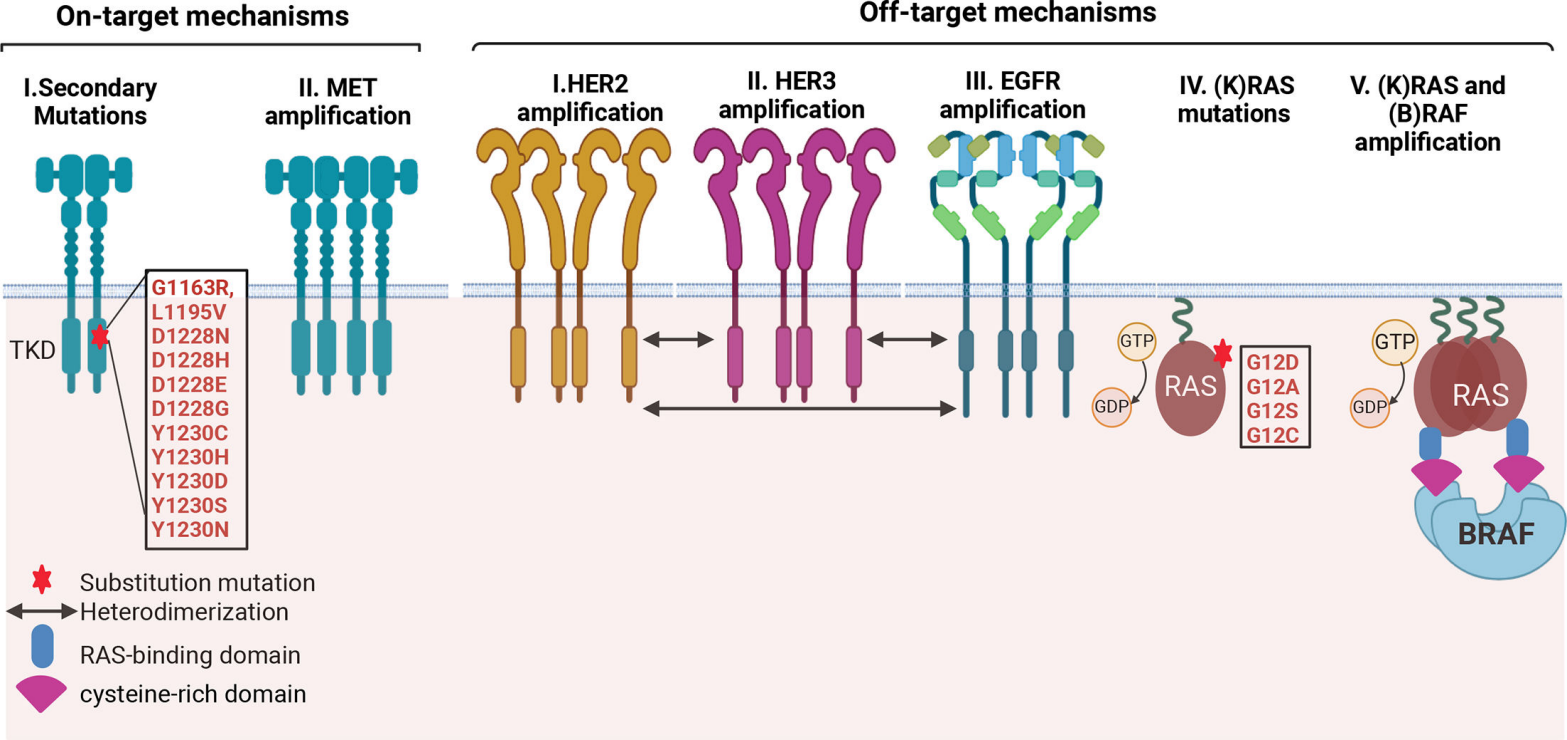
Mechanisms of resistance to MET-targeted therapy in METΔex14 NSCLC. Heterogeneous resistance mechanisms to type-I MET inhibitors. On-target mechanisms of resistance in METΔex14 include alterations in MET structure such as second-site mutations in the tyrosine kinase domain and amplification of MET and METΔex14 allele. Reported off-target mechanisms include amplifications in ERBB family of receptors mainly ERBB2 and 3 (HER2 and 3) which can homo- and heterodimerize between themselves and with EGFR. Amplifications in KRAS and its target, BRAF have also been identified in patients treated with MET-TKIs. TKD, tyrosine kinase domain.

Several MET TKIs and monoclonal antibodies targeting MET are undergoing clinical trials, with some TKIs approved and utilized as targeted therapies in many countries [Table 1]. Although there has been significant improvement in the treatment outcomes of patients treated with MET TKIs, mechanisms such as secondary mutations in the kinase domain, amplification events, and bypass signaling activation can often lead to acquired resistance to targeted therapy [26] [Figure 2]. Independent studies have reported that secondary mutations in the *MET* tyrosine kinase domain, primarily at D1228N/H/E/G and Y1230H/C/D/S/N, confer acquired resistance to crizotinib and other type I MET TKIs while being sensitive to type II MET inhibitors in METΔex14 + NSCLC patients [108–113]. Resistance to type I MET TKIs can also be associated with the amplification of RTKs such as *HER2, HER3*, and *EGFR*, with or without secondary MET mutations [111,114–116]. Although switching to type II TKIs can provide clinical benefits in overcoming resistance to type I TKIs, acquiring secondary mutations and disease progression may still occur [25,115].

**Table 1:** Approved and ongoing clinical trials of METΔex14 cancers

Drug and responsible party	NCT ID-trial name and phase	Number of METΔex14 patients	Dosage	ORR (%)	PFS (month [mo])	Comments
Crizotinib (Xalkori; Pfizer, Inc.) (15,100)	NCT00585195- PROFILE 1001 phase I NCT02499614- METROS phase II	69 26 (9 METΔex14 patients)	250-mg BID, 28-day cycle 250-mg BID, 28-day cycle	32 27	7.3 mo 4.4 mo	Regulatory approval by FDA, not typically the first-line treatment, off-label use, or later-line therapy
Capmatinib (Tabrecta; Novartis Pharmaceuticals Corp.) (24)	NCT02414139- Geometry Mono-1 phase II	Cohort 4: 69 Cohort 5b: 28	400-mg BID, 21-day cycle	Cohort 4: 41 Cohort 5b: 68	Cohort 4: 5.4 mo Cohort 5b: 12.4 mo	FDA-approved for metastatic METΔex14 NSCLC patients
Tepotinib (Tepmetko; EMD Serono Inc.) (101,102)	NCT02864992-VISION phase II	99 (Efficacy population, combined biopsy group)	500-mg OD, 21-day cycle	46	8.5 mo	FDA-approved for metastatic METΔex14 NSCLC patients
Savolitinib (Orpathys; Hutchmed in association with AstraZeneca) (103)	NCT02897479 phase II	70 in total (61/70 tumor response evaluable)	600-mg (≥50 kg) or 400-mg (<50 kg) OD, 21-day cycle	Total: 42.9 tumor response evaluable: 49.2	Not reported	First selective MET inhibitor approved in China. No FDA-approval
Amivantamab and capmatinib combination therapy (Janssen Research & Development, LLC)(104)	NCT05488314- METalmark phase I and II	57 total unresectable (actual METΔex14 patient no. unknown)	Capmatinib: 400-mg oral BID Amivantamab: 700-mg (<80 kg)/1050-mg ( ≥80 kg) IV infusion 28-day cycle and then every 2 weeks from week 5	N/A	N/A	Study active, not recruiting; intended to determine anti-tumor effect of the combination in MET-altered NSCLC (METΔex14 and METamp) patients with stage IV unresectable tumors
TPX-0022 (elzovantinib; Turning Point Therapeutics, Inc) (105)	NCT03993873-SHIELD-1 phase I	95 total (actual METΔex14 patient no. unknown)	Oral administration, 28-day cycle, dosage unknown	N/A	N/A	Study active, not recruiting; intended to determine safety, tolerability, and efficacy of TPX-0022 in advanced/metastatic METΔex14 and MET amplified NSCLC, and other MET-altered solid tumors
APL-101 (vebreltinib; Apollomics Inc) (106)	NCT0317522- SPARTA phase II	497 total, 9 cohorts: 4 cohorts focusing on treatment-naive and treated METΔex14 patients (actual METΔex14 patient no. unknown)	Oral administration, BID, 28-day cycle	N/A	N/A	Study recruiting; intended to assess APL-101 monotherapy for NSCLC and solid tumors with various MET alterations and evaluate APL-101 as an add-on to EGFR inhibitors for NSCLC with acquired MET resistance after EGFR treatment.
Tepotinib in combination with pembrolizumab (Institute of Cancer Research, U.K) (107)	NCT05782361-POTENT phase I	38	Tepotinib 500-mg OD (dose level 1) or 250-mg OD (dose level 1) pembrolizumab 100 mg/vial IV infusion every 21 days with tepotinib	N/A	N/A	Study recruiting; intended to evaluate the efficacy of using a selective MET inhibitor and PD-1 inhibitor in a small group of NSCLC patients with/without METΔex14 who did not respond to systemic therapy or ICIs

BID = twice daily. FDA = Food and Drug Administration. ICI = immune checkpoint inhibitor. IV infusion = intravenous infusion. NCT = National Clinical Trial . OD = once daily. ORR = objective response rate. PD-1 = programmed cell death-1. PFS = progression-free survival. N/A, not available. NSCLC, non-small cell lung cancer.

RAS–MAPK pathway alterations present *de novo* or acquired post-therapy can also affect sensitivity of METΔex14 to MET-TKIs [60,72,117]. Bahcall et al. documented three crizotinib-resistant METΔex14 NSCLC cases in which the resistant tumors exhibited a high copy gain of the WT KRAS allele and discovered a bypass signaling activation via PI3K/AKT restoring resistance to crizotinib upon MEK or KRAS inactivation [117]. Suzawa et al. reported the presence of concurrent KRAS mutant G12D/C/A/S and WT KRAS amplification in NSCLC patients with METΔex14 and demonstrated resistance to both type I and II MET TKIs. The study also established that METΔex14 tumors show higher RAS pathway activation than WT MET-amplified tumors, suggesting greater KRAS tolerance and reliance, which may drive KRAS mutation selection in crizotinib resistance [60].

Expanded use of panel sequencing has facilitated the detection of METΔex14 as a novel post-TKI resistance mechanism in an increasing number of NSCLC patients with other drivers. When METΔex14 coexists with *EGFR* mutations, it most often functions as a nondominant subclone and may contribute to EGFR TKI resistance [69]. Case reports have identified acquired METΔex14 mutation in *EGFR-*mutated NSCLCs where dual targeting of both MET and EGFR was highly effective in regressing lesions and yielding a more durable response [118–121]. Suzawa et al. reported that in isogenic *EGFR*-mutant cells expressing METΔex14, only the combination of osimertinib and crizotinib, rather than either drug alone, was able to inhibit the activation of EGFR, MET, and their downstream effectors, AKT and ERK. The study also established that crizotinib could sensitize the cells to EGFR TKIs [119]. Although dual targeting of EGFR mutations and METΔex14 has demonstrated some clinical benefits in patients, treatment-related adverse events and tolerance, particularly in older individuals, are a concern [122–124]. Acquired METΔex14-mediated resistance to alectinib and brigatinib, selective and multi-kinase inhibitors targeting ALK, has been reported in NSCLC patients with *ALK*-rearrangement [125,126] [Figure 1B]. Combining alectinib and capmatinib conferred a rapid and durable response in a 72-year-old patient with metastatic LUAD harboring *EML4–ALK* fusion gene mutation and acquired METΔex14 [127].

Overall, these findings indicate that resistance mechanisms and clinical outcomes involving METΔex14 are complex and diverse. Effectiveness of IO in METΔex14 tumors indicates that these mutations promote immune evasion and contribute to the development of an immunologically ‘cold tumor.’ A comprehensive understanding of the patient’s genomic profile, treatment history, and PD-L1 status will be important to design an integrated treatment approach to maximize therapeutic efficacy.

**Perspectives**

- Importance of the field: With the current advancement in deep sequencing and *in situ* hybridization technologies, mutations leading to skipping of exon 14 (METΔex14) mutations are now being detected with higher sensitivity both as an actionable driver of non-small cell lung cancer and as a mediator of secondary resistance in other driver-dependent lung adenocarcinomas, rapidly increasing its incidence.
- Current thinking: Given that MET-TKI monotherapy frequently fails to achieve durable responses due to the emergence of acquired molecular alterations and that the clinical benefit of ICIs in METΔex14 remains inconclusive, further investigation into the therapeutic potential of first-line PD-L1 inhibitors, administered either as monotherapy or in combination regimens, is warranted.
- Future directions: Recent studies have reported a high concordance in detecting co-occurring alterations and demonstrated the superior sensitivity of liquid biopsy over tissue biopsy for identifying METΔex14, thereby overcoming challenges of tumor inaccessibility and underscoring the importance of molecular profiling to guide personalized treatment strategies.

## Open Access

This article has been published open access under our Subscribe to Open programme, made possible through the support of our subscribing institutions, learn more here: https://portlandpress.com/pages/open_access_options_and_prices#conditional

## Abbreviations

CHT: chemotherapy
CNS: Central Nervous System
HGF: hepatocyte growth factor
ICI: immune checkpoint inhibitor
IFN-γ: Interferon gamma
IO: immunotherapy
JXD: juxtamembrane domain
LGG: low-grade GBM
LUAD: lung adenocarcinoma
METamp: MET amplification
METΔex14: mutations leading to skipping of exon 14
NGS: next-generation sequencing
NSCLC: non-small cell lung cancer
RTK: receptor tyrosine kinase
TKI: tyrosine kinase inhibitor
TMB: tumor mutational burden
WT: wildtype
Y1003F: Y1003 with a phenylalanine
ctDNA: circulating tumor DNA
nSq: non-squamous
pGBM: primary GBM
sGBM: secondary glioblastoma multiforme
